# G-Quadruplexes in the Regulation of Viral Gene Expressions and Their Impacts on Controlling Infection

**DOI:** 10.3390/pathogens13010060

**Published:** 2024-01-08

**Authors:** Andrew R. Zareie, Prerna Dabral, Subhash C. Verma

**Affiliations:** Department of Microbiology and Immunology, University of Nevada, Reno School of Medicine, 1664 N Virginia Street, Reno, NV 89557, USA; azareie@nevada.unr.edu (A.R.Z.); prerna05dabral@gmail.com (P.D.)

**Keywords:** G-quadruplex, G4, secondary structure, virus regulation, G4 stabilizers, DNA, RNA

## Abstract

G-quadruplexes (G4s) are noncanonical nucleic acid structures that play significant roles in regulating various biological processes, including replication, transcription, translation, and recombination. Recent studies have identified G4s in the genomes of several viruses, such as herpes viruses, hepatitis viruses, and human coronaviruses. These structures are implicated in regulating viral transcription, replication, and virion production, influencing viral infectivity and pathogenesis. G4-stabilizing ligands, like TMPyP4, PhenDC3, and BRACO19, show potential antiviral properties by targeting and stabilizing G4 structures, inhibiting essential viral life-cycle processes. This review delves into the existing literature on G4’s involvement in viral regulation, emphasizing specific G4-stabilizing ligands. While progress has been made in understanding how these ligands regulate viruses, further research is needed to elucidate the mechanisms through which G4s impact viral processes. More research is necessary to develop G4-stabilizing ligands as novel antiviral agents. The increasing body of literature underscores the importance of G4s in viral biology and the development of innovative therapeutic strategies against viral infections. Despite some ligands’ known regulatory effects on viruses, a deeper comprehension of the multifaceted impact of G4s on viral processes is essential. This review advocates for intensified research to unravel the intricate relationship between G4s and viral processes, paving the way for novel antiviral treatments.

## 1. Introduction to G-Quadruplexes

### 1.1. G4 Structure

**Figure 1:**
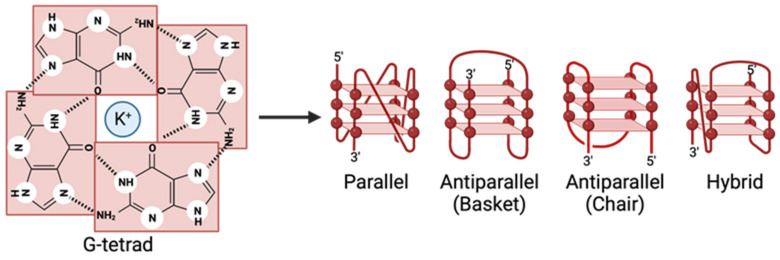
G4 structure. On the left is a G-tetrad and on the right are three G-tetrads stacked to form the different types of G4 structures.

A G-quadruplex (G4) is a noncanonical secondary structure in guanine-rich DNA and RNA sequences. Four successive runs of guanine blocks result in G-tetrads held together by Hoogsteen hydrogen bonding [1,2,3,4]. The hydrogen bonding in G4s differs from the classical Watson–Crick model by the N7 group of guanines interacting with an exocyclic amino group from a neighboring base. The G-tetrads align with a consistent vertical spacing of 3.13–3.3 Å and a twist angle of approximately 30°, forming four grooves. The groove contains solvent molecules adjacent to specific guanine, ribose, and phosphate atoms. Central to the G-tetrads are carboxyl O6 atoms that form an oxygen-based cage and extend through the quadruplex core. The structure is suited for forming a negatively charged channel that can coordinate metal cations in a unique bipyramidal antiprismatic arrangement. A monovalent or divalent cation, such as K^+^, Na^+^, or Ca^2+^, stabilizes the G-tetrad [5,6,7,8]. Potassium is the prevalent stabilizer due to its favorable ionic radius and Gibbs free energy of solvation [9]. In these G-tetrads, guanine bases are connected to ribose sugars via a glycosidic bond χ (N9–C1′), which is constrained to specific dihedral angles, either an anticonformation (ranging from −120 to 180°) or a *syn* conformation (ranging from 0 to 90°). This is determined by the O4′–C1′–N9–C4 arrangement. In DNA’s deoxyribose, both C3′-endo and C2′-endo conformations are possible; in contrast, RNA’s ribose predominantly adopts the C3′-endo conformation. This preference in RNA is attributed to stereoelectronic effects, primarily influenced by the O4′–C1′–C2′–O2′ spatial arrangement [8]. When two or more G-tetrads are present, they can stack upon one another and are stabilized by π–π bonding between the G-tetrads (Figure 1) [10]. The phosphodiester backbone is negatively charged, and the grooves can be targeted by ligands designed for specificity. G4s’ backbone strands are either parallel, antiparallel, or hybrid and form grooves of varying widths, offering diverse structural configurations [11,12,13].

The G4 is classified as parallel when all strands align in the same direction (all up ↑ or all down ↓) [14]. In contrast, antiparallel G4s have strand orientations in different directions (2↑ + 2↓) and, depending on the positioning of the loops, have a chair- or basket-type structure [15]. Hybrid G4s have three parallel and one antiparallel strand (3↑ + 1↓ or 3↓ + 1↑) [13,16]. Three types of loop connections can form in a G4 structure: lateral, diagonal, and propeller. Lateral loops connect antiparallel phosphate backbones that are on the same side of a G4, while cross-diagonal loops link antiparallel strands that are opposite each other on the same G4 surface. Propeller loops connect parallel adjacent strands but on opposite surfaces of the quadruplex. The stability of G4s is proportional to the number of stacked G-tetrads, resulting in higher strength and lower numbers of nucleotides comprising the loop regions, increasing stability [17]. Additionally, the number of G-repeats and the length of the linkers define the G4’s final structure and stability. Short linkers create propeller loops and connect adjacent backbones in a structure with three G-tetrads. Longer linkers form antiparallel alignments [18]. These connecting nucleotides can form base stacking above the G-tetrads, allowing ligand interactions with polycyclic aromatic ring structures. These interactions can cause the G-tetrads to expand, forming larger structures [19].

In human telomeric DNA, intramolecular G4s tend to form two equilibrating hybrid-type structures from two G-tetrads in a K^+^ solution [20,21,22,23,24,25,26,27,28,29]. However, a parallel G4 is formed in the crystalline form and in the presence of a Na^+^ solution, a basket-type G4 [30,31,32]. On the other hand, telomeric repeats composed of RNA adopt a parallel G4 structure [33,34]. Contrastingly to telomeric sequences, the G4s formed in promoter regions often contain more than four G-tetrads and can form multiple complexes. Parallel G4s are the most commonly formed type of G4 in the promoter [30,35]. The topology of RNA G4s is limited to parallel structures due to steric constraints of the 2′-hydroxyl group preventing the *syn*-conformation. Due to restraints on the glycosidic torsion angle, the anticonformation is favored [36,37].

### 1.2. Determining G4 Formation

Computational tools are available for the in silico determination of potential G4-forming sequences (PQSs), such as QGRS Mapper, Quadparser, and PQSFinder [38]. The formation of G4s in cells has been demonstrated by G4-specific antibodies [3,39]. Once the PQSs have been determined, additional techniques can be used to validate if the sequence forms a G4 structure. Some of these techniques include circular dichroism (CD), electrophoretic mobility shift assay (EMSA), nuclear magnetic resonance (NMR), and X-ray crystallography. CD exploits the chirality of G4s, resulting in distinct absorption spectra. Circularly polarized light is passed through a sample, and the different absorption spectra of left- and right-handed light are measured. The peaks from the spectra indicate G4 topology. A parallel G4 shows a positive peak at approximately 260 nm and a negative peak at 240 nm. Antiparallel G4s show a positive peak at 295 nm and a negative peak at 260 nm. The absorption patterns can also be used to study the stability and conformational changes due to ligand binding [40,41]. EMSA utilizes the principle that the electrophoretic mobility of nucleic acid migrates through a native gel differently when its structure is altered. When a G4 structure forms, it migrates faster through a gel than its linear counterpart. This shift in migration is visualized after staining [42]. NMR gives the detailed atomic-level structure and dynamics of G4s. G-rich oligonucleotide sequences are subjected to a magnetic field, and NMR signals are observed. Resonance signals of the NMR spectrum describe the chemical environment of each nucleotide, hydrogen, and nitrogen of the nucleic acids. The signals can be used to determine the positioning of the guanine bases and the overall topology of the G4. NMR can be used to study G4 structural changes over time. Using nuclear Overhauser effect spectroscopy (NOESY), the spatial proximity of the atoms within the G4 can be determined, allowing for three-dimensional structure determination [43,44,45]. X-ray crystallography involves the crystallization of a G-rich oligonucleotide sequence. It is then exposed to an X-ray, and the diffraction pattern is recorded. This technique determines the position of the atoms within the G4 structure, including the formation of the G-tetrads, the coordination of the cation, and the conformation of the loops connecting the tetrads. However, crystallizing G4s can be challenging due to their structural complexity and the conditions necessary to maintain their structure [46,47]. 

### 1.3. G4s in Eukaryotes

**Figure 2:**
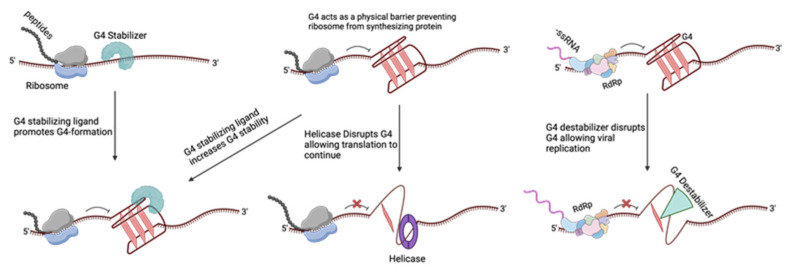
Schematic depicting examples of G4-mediated regulation. Essential steps for viral maintenance and pathogenicity require protein translation and genome replication. The schematic depicts that +ssRNA and G4-stabilizing ligands can promote the formation of G4s in RNA, preventing translation from completing (top left to bottom left). If a G4 is already present, a G4 stabilizer can further enhance the stability of the G4 (top middle to bottom left). Specific proteins can act as G4 helicases and disrupt the structure, allowing translation or replication to continue (top middle to bottom middle). Many RNA viruses require RdRp for viral replication, which can be inhibited by G4s. Certain ligands can act as G4 destabilizers, causing the G4 structure to weaken and revert to a linear form (top right to bottom right).

**Figure 3:**
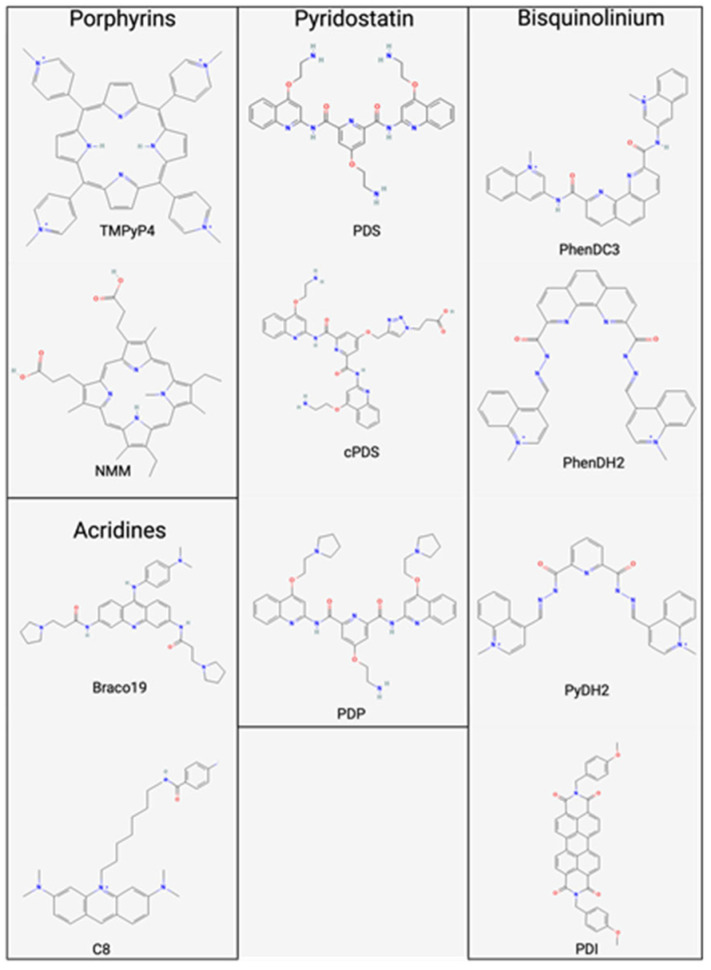
Commonly studied G4-stabilizing ligands. All chemical structures were obtained from PubChem.

G4 structures serve different regulatory roles in both DNA and RNA. G4s serve as regulatory elements in transcription, translation, RNA maturation, and regulating noncoding RNA (Figure 2) [48]. For example, G4s can cause stalling of the RNA Pol II during transcription due to the bulky secondary structure of G4s acting as a physical barrier [49]. Furthermore, there is extensive research on G4s and their role in telomere maintenance [37,50,51]. This review discusses various G4-stabilizing agents (Figure 3) and their roles in modulating the G4s present in viruses. PQSs are prone to adopt a secondary structure in RNA rather than DNA, given RNA’s absence of a complementary strand. This folding propensity contributes to the enhanced stability of the RNA molecule [52]. PQSs are present in functional domains 5′-UTR, ORF, and 3′-UTR. G4s have been shown to affect the coding capacity of a genome through alternative polyadenylation, alternative splicing, and induced frame shifts [48].

Using Quadparser to analyze the human genome, using the pattern G_3+_N_1–7_G_3+_N_1–7_G_3+_N_1–7_G_3+_ identified approximately 376,000 PQSs [53]. The first predicted G4s were within human telomeric single-stranded DNA and later characterized to fold in parallel G4s [1,31]. Telomeric G4s play a role in regulating cellular aging and the unusual processing of telomeres by the human telomerase enzyme. Additionally, these G4 structures have been linked to the development and progression of cancer, suggesting their importance in the cell life cycle and disease [54,55]. G4s within the promoter regions overlapped DNase hypersensitive sites in more than 40% of human genes [56]. The impact of G4s on transcription and translation has been confirmed through experimentation in *cMYC*, *KRAS*, *HRAS*, and *BCL2* [57,58,59,60]. In vitro sequencing studies determined that, in the presence of KCl and PDS, the human genome can form over 700,000 G4s [4]. The G4s in regulatory regions impact gene regulation; however, high-throughput transcriptome-wide expression studies are needed to determine the impact of nonspecific binding of G4-mediated gene regulation.

Several ligands can target G4s to modulate their stability. These ligands show the establishment of a potential therapeutic avenue as antivirals. G4-stabilizing ligands share many characteristics, including polycyclic aromatic scaffolding [61,62,63]. The ligands interact with the G4’s π–π stacking with the terminal tetrad, and the lateral positive charged moieties interact with the phosphate groups within the loops [64]. G4 ligands have the propensity to bind to the upper and lower end positions of the G-tetrads [12]. Modifications to G4-stabilizing ligands have enhanced their selectivity and binding affinity to G4 structures. Given the high conservation of PQSs, the feasibility of a G4-based antiviral therapy is heightened, providing a promising avenue for combating diverse viral strains. Consequently, targeting G4 structures in viruses presents a strategic approach for effective antiviral interventions. 

There have been several published articles surrounding G4s and their ability to regulate viruses. However, due to the constant expansion of scientific knowledge, the rate of new findings requires continual discussion on the progress that has been made. We will be expanding upon and updating works such as those from Ruggerio et al. and Abiri et al., along with introducing newly published G4-based work [65,66,67]. 

## 2. Commonly Studied G4 Ligands

The prospect of utilizing G4s as targets for antiviral therapy stems from their involvement in multiple stages of the viral life cycle, their evolutionary conservation, and their distinct structural characteristics compared to canonical nucleic acid structures. These attributes provide a foundation for developing broad-spectrum antiviral agents with minimal off-target effects on host cells. However, the endeavor to target G4s faces several challenges. First, despite their specificity for G4 structures, there is still a risk of off-target effects on host G4s, potentially resulting in cytotoxicity or other undesirable consequences. Second, the high structural diversity of G4s demands the development of specific ligands tailored for individual G4s in viruses. Lastly, achieving effective intracellular delivery of G4-binding molecules poses a challenge due to the potential for degradation or inadequate cellular uptake. Numerous small molecules have been synthesized and documented for their interaction with G4 structures, showcasing antiviral activity by stabilizing or destabilizing G4s. Some notable examples include those below.

### 2.1. Porphyrins

Porphyrins are a class of heterocyclic macrocycle compounds that interact and stabilize G4s. This class of molecules has a central metal ion surrounded by four pyrrole subunits connected through methine bridges, forming a planar ring [68]. Porphyrins, owing to their strong affinity for G4 structures and their capability to modulate G4-mediated processes, hold promise as potential antiviral agents within this class [69]. One well-studied porphyrin is TMPyP4 (5,10,15,20-Tetrakis-(N-methyl-4-pyridyl)porphine), which is a small molecule shown to interact with and stabilize G4s [70]. TMPyP4’s positively charged porphyrin ring system predominantly binds to the top and bottom of the G4 tetrads, forming electrostatic interactions with the negatively charged phosphate backbone of DNA/RNA [12,71,72]. This stabilization can inhibit the activity of enzymes that would otherwise disrupt or unwind the structure. Studies have shown that the TMPyP4 can selectively bind to and stabilize G4s over other DNA/RNA structures and can inhibit the activity of telomerase, an enzyme that adds telomeric DNA repeats to the ends of chromosomes and is highly active in cancer cells. Additionally, it preferentially binds to parallel G4s over antiparallel G4s [73]. TMPyP4 has been proposed as a potential anticancer agent that can selectively target cancer cells by inhibiting telomerase activity and inducing telomere shortening, leading to apoptosis or senescence [74,75]. TMPyP2 is a structural isomer of TMPyP4 with the N-methyl groups in the two position, causing steric hindrance and preventing binding to the G4, making it an ideal negative control [76]. 

N-methyl mesoporphyrin IX (NMM) is a porphyrin derivative that contains a positively charged porphyrin ring with an N-methyl. The ring can interact with the negatively charged phosphate backbone of DNA/RNA through electrostatic interactions. Specifically for parallel G4s, the N-methyl can fit into the center of the G4 core and align with the stabilizing cation, allowing for efficient π−π stacking [77]. NMM preferentially binds to G4s over duplexed DNA and binds to both parallel and antiparallel G4s [78]. NMM induces the folding of guanine-rich sequences into G4 structures. This has been exploited to develop NMM to detect G4 structures in cells and tissues. NMM is a fluorescent molecule (excitation at λ = 393 nm and emission at λ = 610 nm) when bound selectively to parallel G4s, resulting in up to a 60-fold amplified signal. Since NMM preferentially binds to parallel G4 topology, it can discriminate between different strand orientations based on fluorescent fold enhancement [79].

Like TMPyP4, NMM has been shown to inhibit telomerase activity [80]. NMM has been shown to inhibit the expression of oncogenes and induce the expression of tumor suppressor genes by binding to G4s in the promoter of these genes. This stabilization can prevent transcription factors and RNA polymerase binding to the promoters, thereby inhibiting the oncogenes’ expression and inducing the tumor-suppressor genes [37]. While porphyrins exhibit significant promise as G4-mediated antiviral agents, particular challenges warrant attention. Their relatively nonspecific binding to diverse G4 structures may induce off-target effects on host G4 structures. Additionally, porphyrins’ bioavailability and cellular uptake require optimization to achieve sufficient intracellular concentrations for effective antiviral activity. Nevertheless, porphyrins are a promising class of G4-targeting compounds with potential applications in antiviral therapies. Ongoing research to refine their selectivity, potency, and pharmacokinetic properties is essential in developing safe and efficacious antiviral treatments. 

### 2.2. Bisquinolinium Compounds

Bisquinolinium compounds are small molecules containing bisquinoline moiety, having high affinity and selectivity for G4 structures. PhenDC3 is a well-studied bisquinolinium-derivatized phenanthroline-dicarboxamide that interacts with G4s through extensive π–π bonding between the external tetrads [81]. PhenDC3 has a higher affinity for G4s in nuclear DNA than the predominant duplexed DNA. This compound can organize its internal hydrogen bonds into *syn*-*syn* conformation; this is critical for G4 binding [82]. PhenDC3 binds to G4s through its planar, aromatic perylene ring system. This interaction increases thermal stability, making it more resistant to thermal denaturation or degradation by the nucleases [83]. Like TMPyP4, PhenDC3 has been proposed as a potential anticancer agent, as it selectively targets cancer cells by inhibiting telomerase activity and inducing telomere shortening, leading to apoptosis or senescence [82,84,85]. Additionally, PhenDC3 inhibits the activity of topoisomerase II, a DNA enzyme involved in DNA replication and transcription [84,86]. Much like porphyrins, bisquinolium compounds carry the potential for off-target effects. Nevertheless, through ongoing refinement efforts, bisquinolium compounds exhibit promising potential as G4-mediated antiviral agents.

### 2.3. Napthalene Diimides

Napthalene Diimides (NDIs) are a class of organic molecules that exhibit high affinity for and stabilize G4s. NDIs have a flat, planar structure that allows them to stack into the planar guanine bases of G4 structures [87]. The enhancement of the G4’s overall polarity results from the *π*–*π* stacking interaction between the planar naphthalene core of NDIs and the G-tetrads, along with the imide groups engaging with the loops and grooves within the G4 structure. The increased polarity consequently results in heightened structural instability. The interaction between NDIs and G4s is further stabilized by hydrogen bonding and Van der Waals forces [87]. With remarkable stability, the G4–NDI complex is valuable for diagnostic and therapeutic applications. Developing derivatives with heightened G4 affinity and selectivity further enhances their potential. Specifically, these compounds demonstrate a strong binding capacity to G4s in telomeres and oncogene promoters, rendering them particularly advantageous for anticancer research [61,62,63,88]. NDI derivatives have been developed to improve G4-binding affinity, selectivity, and pharmacokinetic properties. 

### 2.4. Acridines

The N, N′-(9((4(dimethylamino)phenyl)amino)acridine-3-6-diyl)bis(3-(pyrrolidine-1-yl)propenamide), commonly known as BRACO19, is a 3,6,9-trisubstituted acridine compound that stabilizes G4 structures. The 3,6,9-trisubstituted acridine compounds have a high selectivity for G4s over duplexed DNA, lower cytotoxicity, and inhibit telomerase activity [89,90,91]. This interaction is based on a central planar pharmacophore that facilitates asymmetrical binding with guanine bases of the G-tetrads via *π*–*π* interactions and the nitrogen atom of the acridine ring aligns with the K^+^ cations within the ion pore [92,93]. The two side chains containing tertiary amine moiety allow recognition of the grooves in G4 structures [89]. BRACO19 can bind G4s through top stacking, bottom intercalation, and groove binding [94]. This acridine derivative has effective antitumor activity, including epidermoid carcinoma, prostate cancer, uterus carcinomas, and flavopiridol-resistant colorectal cancer HCT-116 cells [95,96,97,98,99].

### 2.5. Pyridostatin/Derivatives

Pyridostatin (PDS) was designed to have an electron-rich, flat, aromatic G4-stabilizing ligand that is able to participate in hydrogen bonding. Through G4 stabilization, PDS inhibits telomeric elongation and triggers a DNA damage response to the telomeres [100,101]. It also increases telomere fragility, reducing the proliferation of homologous recombination by causing double-stranded breaks and deregulating G2/M progression [102]. Utilizing the PDS scaffold has created many derivatives capable of inhibiting cancer cell replication through G4-mediation [103]. PDS has been used in next-generation sequences because it induces polymerase stalling. It has aided in the identification of over 716,000 DNA G-rich sequences in the human genome [4]. RNA G4 sequencing, in conjunction with PDS, helped create a global in vitro map of RNA G4s that were formed and determined RNA G4-dependent differences in RNA folding [104].

## 3. Alternative Strategies and Ligands in Targeting G4s

Strategic therapeutic approaches harnessing the unique structural attributes of G4s seek to pioneer innovative strategies for combating viral infections. By intricately targeting and manipulating the distinct features of G4s, researchers aim to develop novel antiviral agents that selectively interact with these specialized nucleic acid arrangements. This tailored interaction is anticipated to modulate critical viral processes, including replication, transcription, and translation, ultimately leading to the suppression or eradication of the virus. However, a critical consideration of these strategies is the potential for off-target effects, such as inadvertent binding to host G4s, possibly resulting in cytotoxicity or other unintended adverse effects. Furthermore, the delivery of many of these strategies into target cells presents a formidable challenge, as they may be susceptible to degradation or insufficient uptake by cells, potentially limiting their efficacy [105].

Although many G4-stabilizing ligands cannot differentiate between viral and human G4 structures, there are some possible explanations as to why there are significant antiviral effects seen. The first is the sheer number of virions released from infected cells, which is estimated to be in the thousands [106]. The number of PQSs that form G4s at a given moment in humans is about 10,000 [107,108,109,110]. The number of virions released per cell, 1000 (for HSV-1), and the number of PQSs they have far outweigh that of the human genome [104,111]. Additionally, by using immunofluorescence to measure the G4 signals, it has been determined that G4s are significantly higher in infected cells [112]. Therefore, by targeting infected cells, the G4s that would be stabilized have a higher likelihood of being viral G4s over host G4s. Second, host G4s have host proteins interacting with them and can be chromatinized, whereas viral G4s lack these interactions. Third, viruses require the host machinery for replication and do so by manipulating the host’s cell cycle [113]. PQSs are present in genes associated with the cell cycle and gene promoters for transcription [54,114,115]. G4 ligands can, therefore, transcriptionally modulate those genes, allowing the cell to be less permissive and increasing antiviral activity. Last is consideration of the localization of the G4s within the cell. RNA viruses maintain themselves in the cytoplasm, so G4 ligands that localize to the nucleus can only have indirect antiviral activity, as previously described [116]. 

### 3.1. Antisense Oligonucleotides 

Antisense oligonucleotides (ASOs) have risen as a promising therapeutic avenue for targeting viral G4s, owing to their exceptional specificity and capacity to regulate gene expression. These succinct, synthetic DNA/RNA molecules are meticulously designed to bind with high precision to complementary target RNA sequences, specifically those within PQSs in the viral genome. This targeted binding enables the meticulous manipulation of viral gene expression, presenting a distinctive opportunity for developing groundbreaking antiviral therapies [117]. Upon hybridization with the target RNA, ASOs disrupt G4 structures, interfering with RNA folding and preventing the formation of stable, functional G4s. This disruption affects various aspects of viral gene expression, including transcription, translation, and post-transcriptional modifications, ultimately impacting viral replication and pathogenesis [117]. For example, ASOs can be used to disrupt a G4 structure within Epstein–Barr virus-encoded nuclear antigen 1 (EBNA1). EBNA1 is essential for viral gene regulation, extrachromosomal replication, and maintenance of the EBV episome [118,119]. This prevents the virus from autoregulating EBNA1’s expression and increases translation. This causes an increase in antigen presentation, allowing for host immune detection [120]. Therefore, ASOs are a potential strategy to disrupt the formation of viral G4s. This emphasizes the need to understand viral G4 interaction. 

ASOs offer several advantages over traditional antiviral therapies, including high specificity for viral target sequences, the ability to target multiple G4 structures simultaneously, and a reduced likelihood of developing resistance due to their unique mode of action [121]. Additionally, ASOs can be chemically modified to improve stability, binding affinity, and pharmacokinetic properties, further enhancing their therapeutic potential [122].

### 3.2. CRISPR–Cas9-Mediated G4 Stabilization

The CRISPR–Cas9 system has emerged as a potent genome-editing tool with the potential to be repurposed for targeting and stabilizing G4s within viral genomes [123]. Utilizing guide RNAs (gRNAs) specifically designed to recognize PQS regions, CRISPR–Cas9 can introduce targeted mutations or insertions that facilitate G4 stabilization. Targeting G4s with CRISPR–Cas9 can offer multiple advantages, such as high specificity and efficiency, along with the potential to target multiple G4 structures simultaneously by employing an array of gRNAs designed to recognize distinct PQS regions within the viral genome [124]. The versatility of the CRISPR–Cas9 system also enables the investigation of various G4-mediated regulatory mechanisms by generating targeted modifications within the viral genome. Targeting stem-loop 3 of the gRNA and substituting C76G/A68G or 60 + G/75 + G results in an inducible G4 site. Therefore, when CRISPER–Cas9 binds to a sequence when PDS or PDP is added, a G4 forms, causing a regulatory effect [125]. Therefore, in the case of viruses, specific regions in the viral genome can be targeted to inhibit the replication process. Further research into optimizing gRNA design, delivery methods, and potential off-target effects will be essential in harnessing the potential of the CRISPR–Cas9 system for G4-based antiviral therapy.

### 3.3. RNA Interference and Small Interfering RNA 

RNA interference (RNAi) is a highly conserved mechanism within cells that has a critical function in regulating gene expression using small RNA molecules, such as microRNAs and small interfering RNAs [126]. The process involves introducing small RNA molecules into the RNA-induced silencing complex (RISC), which then binds to the target mRNA sequences causing either cleavage or translational repression, ultimately resulting in post-transcriptional gene silencing [127]. RNAi-based therapies offer several advantages over traditional antiviral treatments, including high specificity for target sequences, reduced likelihood of developing resistance due to the unique mode of action, and the ability to target multiple G4 structures simultaneously [128]. Additionally, RNAi can be tailored to target highly conserved regions within viral genomes, potentially enabling the development of broad-spectrum antiviral agents [129]. As an effective therapeutic approach, RNAi targets G4s in viral genomes by degrading complementary messenger RNAs selectively and efficiently, reducing the replication of viruses [130,131]. Targeting the PQSs responsible for forming G4s decreases gene expression levels, ultimately leading to the post-transcriptional silencing of genes via mRNA cleavage or translational repression through incorporation into the RISC complex [132,133].

### 3.4. Aptamers

Aptamers are oligonucleotides composed of short, single-stranded DNA or RNA with unique three-dimensional configurations capable of specifically and tightly binding to target molecules. These nucleic acid-based agents, also known as “chemical antibodies”, can recognize a broad range of targets, including proteins, small molecules, and nucleic acids [134]. Leveraging this versatility, aptamers emerge as a promising strategy for antiviral interventions, showcasing the potential to disrupt viral propagation by specifically targeting viral G4s [135]. By attaching themselves to viral G4s or interfering with the interactions between viral proteins and G4s, aptamers can disrupt vital processes for viral persistence. 

Aptamers are promising antiviral agents due to their remarkable specificity and high affinity for targets, minimizing the off-target effects on host cells. Their synthesis and modification are relatively straightforward, facilitating rapid development and optimization. The systematic evolution of ligands by exponential enrichment (SELEX) process allows for the selection of aptamers with optimal binding characteristics for a given target [135,136]. G4s themselves can be aptamers, and when used in conjunction with nanoparticles, G4s enhance anticancer drugs’ loading capacity and amplify their biological effects. A notable example is AS1411, an aptamer designed to recognize Nucleolin (NCL), a multifunctional DNA/RNA binding protein overexpressed on the surface of diverse cancer cell types, including B-cell chronic lymphocytic leukemia, breast cancer, cervical cancer, gastric cancer, etc. [137,138,139,140,141]. TMPyP4 is crucial in photodynamic therapy, demonstrating efficacy in treating malignant and premalignant tissues. Upon excitation by optimal light, TMPyP4 generates singlet oxygen in cancer cells, inducing cell death [142]. When combined with AS1411, TMPyP4 exhibits a 3.8-fold increase in accumulation in breast cancer cells compared to normal fibroblasts and epithelial cells [143]. AS1411 has been used as a transporter for various compounds, including acridines, phthalocyanines, and porphyrin derivatives [143,144,145]. An array of AS1411 derivatives has been synthesized, and ongoing research has been conducted to increase their delivery specificity/uptake, anticancer properties, and greater nuclease resistance [139,146,147]. While several AS1411 derivatives show promise, further testing is required, and the development of additional G4 aptamers is also underway. This concerted effort underscores the potential for advancing targeted therapies in cancer treatment [148,149]. 

## 4. DNA Viruses

DNA viruses are a diverse group of viruses with single or double-stranded DNA genomes. They can have circular or linear DNA and vary in size. Unlike RNA viruses, DNA viruses replicate their genome within the host cell’s nucleus, allowing for more complex replication strategies [150]. Following the import of the viral genome into the nucleus, it is then transcribed by the host’s transcription machinery, producing viral mRNA that is translated by host ribosomes into viral proteins. These proteins, along with the replication of the viral genome, assemble into new virion particles. The replication of DNA viruses within the host cell’s nucleus additionally allows for the establishment of latent infections, where the viral genome is maintained in a dormant state within the host cell [151].This is seen in herpesviruses, which can establish lifelong infections in the host and reactivate to cause disease later in life [152].

DNA viruses exhibit diverse replication mechanisms based on their virus family. For instance, herpesviruses employ a rolling circle mechanism, replicating viral DNA as a long, single-stranded molecule that cleaves into unit-length genomes. In contrast, adenoviruses utilize a strand displacement mechanism, replicating viral DNA as a double-stranded molecule with a displaced strand serving as a template for further replication. Additionally, DNA viruses possess protein capsids that encapsulate the genome, with variations in capsid shape, including icosahedral or helical forms. Some of these viruses are enveloped and feature a lipid membrane surrounding the capsid, as observed in the case of herpesvirus [153]. 

### 4.1. Herpesviridae

Human herpesviruses (HHVs) cause life-long infections that can lead to many diseases. All HHVs have large dsDNA genomes enriched in GCs and PQSs [154]. Herpesviruses are divided into three subfamilies: alpha, beta, and gamma herpesviruses. Alpha herpesviruses include the herpes simplex virus 1 (HSV-1), responsible for oral infections leading to sores, fever, or blisters; the herpes simplex virus 2 (HSV-2), responsible for genital herpes infections; and the varicella-zoster virus (VZV) causes chickenpox (varicella) in the initial infection and shingles (zoster) upon reactivation [155]. 

**Figure 4:**
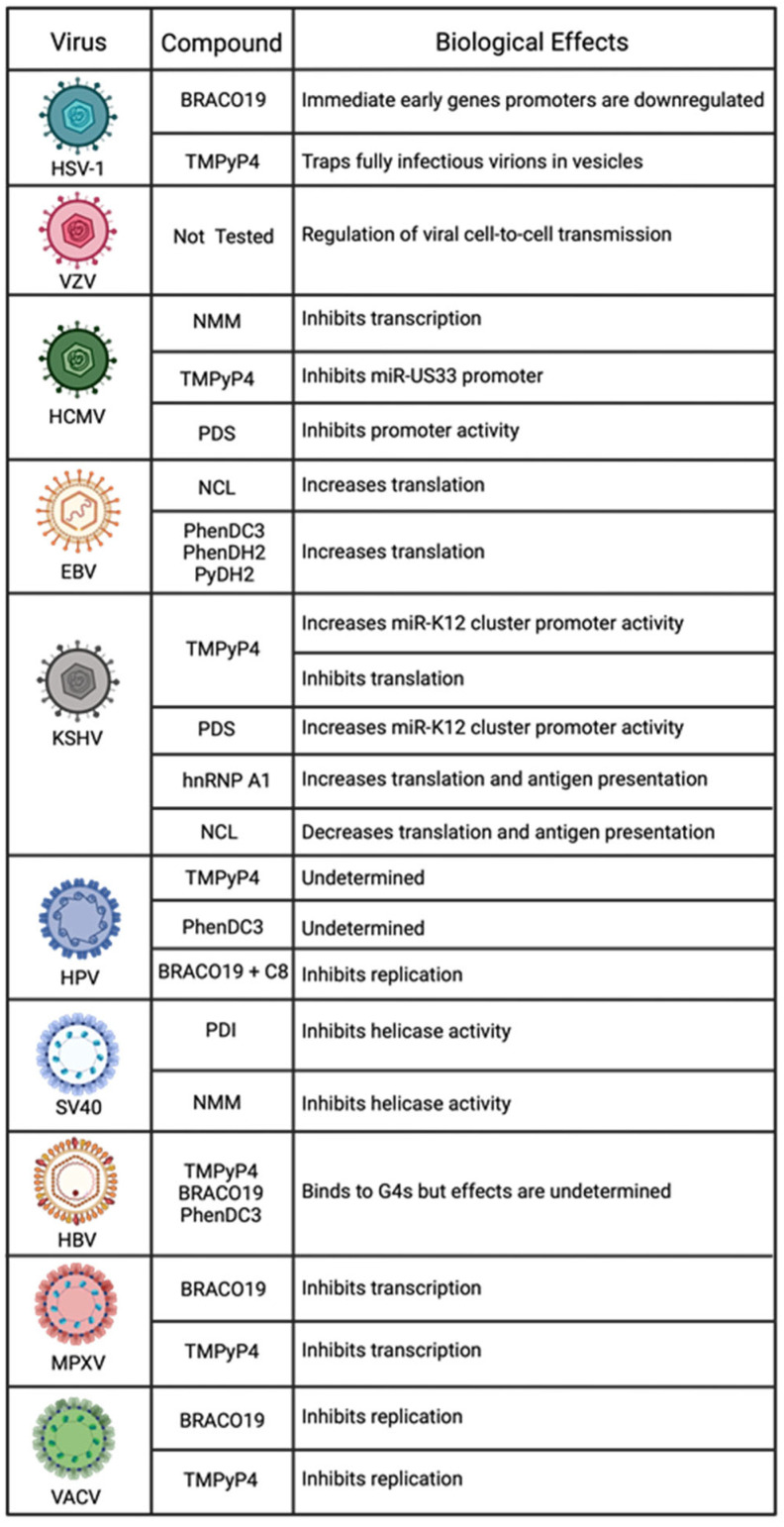
Summary of DNA viruses and the modulatory effects of the G4-stabilizing compounds that regulate them.

HSV-1 contains abundant PQSs in the repeat region of the viral genome. Specifically, G4s have been found in the promoters of several HSV-1 genes, including the immediate early genes ICP0 and ICP4. ICP0 and ICP4 are essential for HSV-1 replication, resulting in high regulation. G4s in promoter regions of these genes can regulate transcription by inhibiting the binding of transcription factors or blocking the access of RNA polymerase to the promoter region [156]. ICP4 positively autoregulates its promoter through a G4 interaction. However, BRACO19 can stabilize this G4, causing transcriptional downregulation of ICP4 [157]. Similarly, quindoline derivatives displayed a nanomolar-range anti-HSV-1 activity, inhibiting ICP4 expression [158]. Therefore, these two compounds can inhibit HSV-1’s viral replication (Figure 4). 

TMPyP4 binds to and stabilizes a G4 in HSV-1’s repeat regions. Interestingly, TMPyP4 does not affect either HSV-1 viral entry or replication; however, it does trap fully infectious virions in vesicles, which is independent of the autophagy process [159]. Furthermore, TMPyP4 interacts with HSV-1 G4 structures, inhibiting polymerase progression in vitro and exhibiting substantial antiviral activity in infected cells [159]. Examination of HSV-1 immediate early (IE) genes reveals that adding BRACO19 increases the melting temperature, confirming G4 stabilization through CD thermal unfolding analysis. Utilizing the luciferase firefly gene cloned downstream of the promoter of two HSV-1 IE genes, treatment with BRACO19 affected the activity of both promoters in a dose-dependent manner. Stabilizing the HSV-1’s G4 sites results in the inhibition of IE transcription [160]. Additionally, the gp054 gene, responsible for encoding UL36, a critical component of the tegument protein, contains a G4 that can be stabilized by BRACO19, resulting in transcriptional regulation of UL36. Lastly, BRACO19 impedes the processing of Taq polymerase at sequences that form G4 structures in the HSV-1 genome, reducing intracellular viral DNA levels within infected cells [159]. 

The investigation of VZV revealed an abundance of G4 motifs in the internal and terminal repeat short regions. A notably high concentration of G4 motifs is present on the template strand within ORF14, responsible for encoding glycoprotein C (gC), which is a virulent agent essential for viral growth in skin cells. G4 formation through the reiteration sequence results in suppressed gC expression during VZV infection and results in the regulation of viral cell-to-cell transmission [161]. 

Beta herpesviruses include human cytomegalovirus (HCMV), which can lead to severe infections in immunocompromised individuals and is the leading cause of congenital abnormalities; human herpesviruses 6A and 6B (HHV-6A and HHV-6B), associated with roseola infantum, a common childhood illness characterized by a high fever and rash; and human herpesvirus 7 (HHV-7), linked to febrile illnesses that primarily affects young children [162,163,164]. Analyzing 36 PQSs within 20 genes of HCMV showed that, in the presence of G4-stabilizing ligands, nine genes were affected by the G4 formation/stabilization by NMM in HCMV gene promoters [83]. The application of NMM on cells caused a significant inhibition in the expression of UL76, likely due to its impact on GQ18 located within the promoter. NMM stabilized parallel G4s in HCMV but did not affect antiparallel G4s. Additionally, these PQSs were stabilized by TMPyP4, increasing their stability while not affecting their conformations [83]. HCMV has a PQS motif upstream of the microRNA cluster miR-US33 promoter. Viral miRNAs are essential for maintaining viral latency and are heavily dependent on the presence of viral miRNAs, which exert their inhibitory effects on host genes and viral modulators [165]. TMPyP4 destabilizes miRNA G4s for HCMV, inhibiting miR-US33 promoter activity, while PDS has the opposite effect by stabilizing miRNA G4s in HCMV [166]. 

HHV-6A/B integrates its genome into the telomeres of chromosomes [167,168]. Incubating BRACO19 into telomerase-expressing cells modifies the conformation and further stabilizes the G4 structures in telomeric DNA. This causes a drastic reduction in HHV-6A’s ability to integrate into the host’s telomeres [169]. This exemplifies how stabilizing compounds can target G4s in human sequences, resulting in diminished viral capacity.

Gamma herpesviruses include the Epstein–Barr virus (EBV), which causes infectious mononucleosis and is associated with different cancers, including Burkitt’s lymphoma, nasopharyngeal carcinoma, and Hodgkin’s lymphoma and Kaposi’s sarcoma-associated herpesvirus (KSHV), which is associated with Kaposi’s sarcomas, primary effusion lymphoma, and multicentric Castleman’s disease [170]. The G4s in EBNA1 mRNA modulate viral translation. Destabilizing the G4s in EBNA1 using antisense oligos complementary to EBNA1’s glycine–alanine repeat domain (GAr) mRNA results in enhanced translation and antigen presentation on the cell surface. However, the opposite is also true; stabilizing EBNA1’s GAr results in decreased translation and antigen presentation [120]. NCL binds with the GAr region of EBV’s EBNA1 mRNA, leading to a consequential downregulation of EBNA1 expression. Intriguingly, PhenDC3 exhibits a higher affinity for these G4 structures than NCL, thereby promoting the increased translation of EBNA1 and enhancing antigen presentation [171]. Compared to PhenDC3, PyDH2 and PhenDH2 demonstrated an improved capacity to enhance EBNA1 expression with reduced toxicity. These ligands exhibited the ability to disrupt the NCL-EBNA1 mRNA interaction, ultimately facilitating enhanced antigen presentation [172]. 

It is important to note that the precise positioning of G4s in RNA is crucial for their activity. As Lista et al. (2017) proposed, the interaction between NCL and G4s within EBNA1 mRNA is sufficient to impede the synthesis of both antigenic peptides and complete proteins [171]. A recent study by Zheng et al. (2022) elucidated that the positioning of EBNA1 GAr mRNA at either the 5′ or 3′ end of the chicken ovalbumin (Ova) open reading frame (ORF), leading to the formation of N or C terminus GAr–OVA and OVA–GAr fusion proteins, resulted in distinct expression levels. Notably, NCL exhibited nearly double the binding affinity to GAr–Ova compared to Ova–GAr. This emphasizes that the precise location of GAr G4s within the mRNA coding sequence is a crucial modulator of NCL binding and translation inhibition [173]. 

In Kaposi’s sarcoma-associated herpesvirus (KSHV), PQSs were identified upstream of the miRNA cluster miR-K12p1-9-11 promoter. Treatment with TMPyP4 destabilized miRNA G4s, enhancing the KSHV miR-K12 cluster promoter activity [174]. Furthermore, PDS also stabilizes miR-K12p1-9-11 promoter similarly to TMPyP4. Additionally, TMPyP4 stabilized G4 structures identified in the latency-associated nuclear antigen (LANA) [122,175], the protein most expressed during latency and fundamental for viral transmission and host immune surveillance evasion [176,177,178,179]. The addition of TMPyP4 resulted in a decrease in the levels of LANA within cells infected with KSHV. This observation provides corroboration for the hypothesis that enhancing G4 stability within LANA mRNA prompts the inhibition of translation processes. Fortifying G4 structures in LANA mRNA led to diminished surface expression and presentation of the corresponding antigen amongst KSHV-positive cells, thereby aiding in maintaining viral infection [175]. Conversely, heterogeneous nuclear ribonucleoprotein A1 (hnRNP A1) has been demonstrated to modulate various biological processes by interacting with G4 structures [180]. This interaction involves the selective binding of hnRNP A1 to the guanine-rich region of mRNA, consequently leading to the unfolding of G4 structures [180,181]. hnRNP A1 functions as a helicase, destabilizing the G4 secondary structure, leading to increased LANA translation and enhanced antigen presentation [175]. Contrastingly, NCL stabilizes the same G4 hnRNPA1 unwinds, resulting in reduced LANA translation and reduced antigen presentation [182]. Therefore, if this interaction can be destabilized, antigen presentation is increased, allowing the host immune system to detect and eliminate infected cells. 

G4s in KS-Bcl-2 and BHRF1 significantly enhanced vBcl-2 expression, with PDS stabilizing the G4 in their promoters, resulting in increased promoter activity [183]. In the terminal repeat (TR) of KSHV, PhenDC3 stabilized G4s, causing replication fork stalling and reducing viral DNA replication. Additionally, PhenDC3 decreased overall genome copies, leading to the eventual loss of the viral episome in infected cells [184]. These findings underscore the intricate regulatory roles of G4s in KSHV, offering potential targets for therapeutic interventions.

### 4.2. Human Papillomavirus

Human papillomaviruses (HPVs), a double-stranded DNA virus, are a prevalent sexually transmitted infection that can result in the onset of skin, head, and neck, as well as anogenital, cancer. Among these cancers is cervical cancer, which poses one of the most crucial global health issues. Only 8 out of 120 known HPVs contain G4s, and there are only seven PQSs located in the long control region (LCR), structural protein L2, early region E1, and E4 regions of the HPV genome [185,186]. Incubation of TMPyP4 with the G4s identified in the seven HPV strains reported high G4 stabilization, assessed by FRET and CD melting experiments [187]. PhenDC was also confirmed to effectively stabilize HPVs’ G4s in vitro. Despite encouraging in vitro studies, the compound was unsuccessful in reducing viral replication and protein expression when tested on organotypic raft cultures [187]. However, BRACO19, along with C8, an acridine derivative, was found to have a high affinity to HPV G4s, with a high affinity to a fluorescent intercalator displacement assay (G4-FID). The antiviral activity of C8 was assessed in organotypic epithelial cultures infected with HPV16 or HPV18. The results showed that administering C8 for 20 days effectively decreased viral replication. As current treatments cannot eliminate HPV due to latent reservoirs, targeting G4s within the viral genome presents a potential approach for addressing both active and latent viruses [187].

### 4.3. Polyomavirus

Polyomaviruses are nonenveloped, double-stranded DNA viruses that belong to the Polyomaviridae family. These viruses have been associated with various diseases in humans and animals, including tumors, nephropathy, and progressive multifocal leukoencephalopathy (PML) [188,189]. Some well-known members of the Polyomaviridae family include the JC virus (JCV), BK virus (BKV), and simian virus 40 (SV40). Polyomaviruses possess a circular, double-stranded DNA genome comprising about 5000 bp. This genetic material is partitioned into three sections: the early, late, and noncoding control regions (NCCR). The early region encodes vital viral regulatory proteins such as large T-antigen and small t-antigen, which are indispensable for viral replication and transformation. Meanwhile, structural proteins like viral capsid proteins VP1, VP2, and VP3 are encoded in the late section of the genome [190]. Additionally, NCCR holds within it both the origin of replication and regulatory elements that monitor viral transcriptional activity alongside its overall duplication process [191].

NCRR in SV40 is a crucial element that not only contains the ORI and encapsulation sequence but also regulates transcription direction. The presence of six GC boxes with repeated GGGCGG sequences forms an unusual quadruplex structure, which NMR has determined to contain a C-tetrad stacked between two G-tetrads [192]. These GC-rich motifs act as binding sites for SP1 and significantly involve early transcription. However, replication of the SV40 genome necessitates TAg, a multifunctional protein that interacts with p53 and Rb besides binding to ORI and possessing adenosine triphosphate-dependent helicase activity [193]. Notably, TAg can unravel G4 DNA structures, thereby playing an imperative role in controlling both early/late transcription and replication [194]. Inhibiting both the duplex DNA helicase activities of TAg alongside destabilizing G4s is feasible using Perylene di-imide derivatives (PDI) or NMM. Notably, TE111, TE101, and PIPER effectively stabilize the G4 structure, inhibiting Tag helicase activity. NMM and coralyne interact with the loops and grooves of the G4, resulting in adequate G4 protection. On the other hand, end stackers such as TMPyP4 are ineffective at inhibiting TAg helicase activity [195]. 

### 4.4. Hepatitis B 

Hepatitis B virus (HBV) is a small, partially double-stranded DNA virus from the Hepadnaviridae family. HBV is responsible for acute and chronic hepatitis infections that can lead to liver disease, including cirrhosis and hepatocellular carcinoma [196]. A G4 motif, which is highly conserved, was identified in the promoters of the preS2/S gene. This motif forms a hybrid intramolecular G4 structure, which regulates transcription and virion secretion in HBV genotype B [197]. More recently, a G4 structure in the precore promoter of the HBV genome is conserved in nearly all HBV genotypes. This is essential for generating pregenomic RNA, synthesis of core and polymerase proteins, and genome encapsidation [198]. TMPyP4, BRACO19, and PhenDC3 have been shown to bind to G4s in HBV [199]; however, research is still ongoing to see if these interactions result in regulating HBV infectivity. 

### 4.5. Monkeypox Virus

The monkeypox virus (MPXV) is a double-stranded DNA virus belonging to the Orthopoxvirus genus within the Poxviridae family. MPXV is closely related to the smallpox virus and is endemic in parts of West and Central Africa. In 2022, MPXV emerged in nonendemic regions, leading to a public health emergency of international concern being declared by the WHO. Initial symptoms include fever, headache, swollen lymph nodes, and the development of pox-like marks on the body [200]. A total of 86 PQSs have been identified in MPXV. In a primer extension assay, it was found that BRACO19 and TMPyP4 stabilized a G4 site in MPXV, thereby inhibiting the completion of transcription. Additionally, the vaccinia virus (VACV) has a genome highly similar to MPXV. In VACV, BRACO19 and TMPyP4 suppressed viral replication [201]. These initial results indicate that orthopoxviruses may be able to be regulated with BRACO19 and TMPyP4 to suppress viral transcription and replication. 

## 5. RNA Viruses

RNA viruses comprise a major class of pathogens with substantial implications for human health and disease. Their genomes are composed of single-stranded RNA. Replication occurs within the host cell’s cytoplasm, facilitated by a virus-encoded RNA-dependent RNA polymerase (RdRp) responsible for copying their RNA genome [202]. Another significant difference between RNA and DNA viruses is that RNA viruses tend to mutate faster than DNA viruses due to the lack of proofreading activity in their RNA polymerase, leading to higher rates of mutations [203]. This rapid evolution is a factor that allows RNA viruses to adapt to new hosts and environments, contributing to their ability to cause emerging infectious diseases. RNA viruses can be categorized based on whether they have positive-sense or negative-sense RNA. Positive-sense viruses have RNA that functions like mRNA and can be immediately translated into proteins by the host cell’s machinery. Contrastingly, negative-sense RNA viruses have RNA complementary to mRNA and must first be transcribed into positive-sense RNA before translation can occur [204]. 

### 5.1. HIV

The human immunodeficiency virus (HIV) was first recorded in 1983 [205,206,207]. HIV can maintain latency for over a decade before reactivation, resulting in acquired immunodeficiency syndrome (AIDS). As of 2018, the CDC reports 1.7 million new cases of HIV, resulting in a total of 37.9 million infected worldwide. Since 1981, over 35 million people have died, and approximately 750,000 die annually [208]. HIV surfaced through the multi-independent zoonotic transmission of the simian immunodeficiency virus, changing host from primates to humans [209,210]. It is a lentivirus, part of the Retroviridae family, and utilizes reverse transcription (RT) and integrates its viral genome into the host’s. This allows for a persistent and lifelong infection [211]. 

G4 structures were identified and confirmed using CD in long terminal repeat (LTR) 1 and 3 and the Nef gene [212,213]. LTRs serve as viral promoters and have regulatory enhancer/suppressor regions [214,215]. G4s in HIV promote dimerization of the two viral RNA strands when encapsulated by the nucleocapsid. This allows RT to strand-hop if there are breaks in the template. This facilitates the successful completion of RT, subsequently allowing the integration of the viral genome into the host’s genome [216,217]. In addition, strand hopping promotes genetic diversity between strains, which can lead to resistance to different antiretrovirals [218]. Nef is essential for viral replication and pathogenesis in early infection [219]. Nef RNA can evade interfering RNA with alternative folding and prevent downregulation. HIV encodes its own miRNA (miR-N367) that targets for self-transcriptional regulation [220]. 

**Figure 5:**
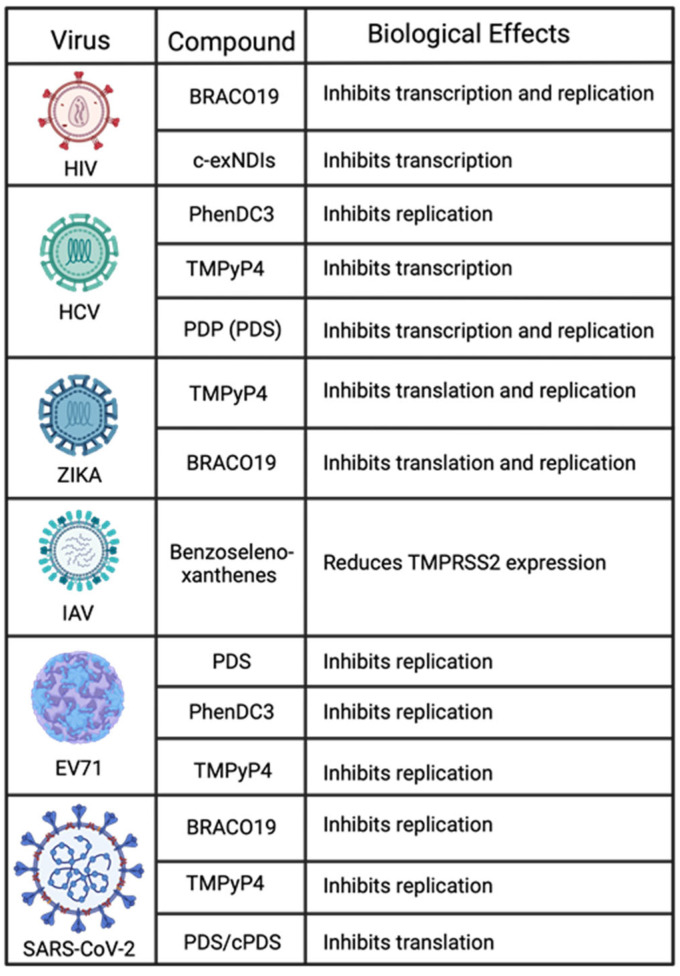
Summary of RNA viruses and the modulatory effects of the G4-stabilizing compounds that regulate them.

C-exNDIs were designed to be selective of viral G4s [62]. Luciferase reporter assays using HIV LTR and mutated LTR, without G4 secondary structures, were cloned upstream of the firefly luciferase plasmid. Indirect transcriptional repression was measured by the translational output of luciferase intensity between G4-stabilizing ligands BRACO19 and c-exNDIs. TMPyP2, a non-G4-stabilizing porphyrin, was used as a control to ensure the presence of a bulky G4 ligand, but it did not result in G4 stabilization. Previous results have shown that G4 ligands can induce G4 assembly [213]. Both inhibited HIV-1 viral activity by interacting with the G4s in the LTR region (Figure 5). Looking at their respective data, BRACO19 has up to 50% HIV inhibition prehost integration and 80% postintegration, whereas c-exNDIs have up to a 65% reduction postgenome integration [62]. 

### 5.2. Hepatitis C

The Flaviviridae family includes the small, enveloped, and single-stranded RNA virus known as Hepatitis C (HCV), which has a global impact on public health. HCV predominantly targets the liver, causing chronic hepatitis, hepatocellular carcinoma, and cirrhosis [221]. While direct-acting antiviral drugs have transformed treatment options for HCV patients, drug resistance remains problematic, along with inadequate accessibility to therapy and a lack of an efficacious vaccine. The genome of HCV spans approximately 9.6 kilobases and is composed of a solitary ORF flanked by untranslated regions at the 5′ and 3′ ends [222]. The ORF encodes for a polyprotein that undergoes co- and post-translational processing to produce no less than ten viral proteins, comprising structural proteins such as core, E1, and E2, and nonstructural ones like p7, NS2, and NS5B. Within the 5′ UTR lies an internal ribosome entry site (IRES), responsible for facilitating cap-independent translation of the viral polyprotein, while the essential replication components lie in the contiguous 3′ UTR region necessary for assembling virions [223]. A recent study showed that PhenDC3 could hinder HCV’s RdRp and inhibit viral replication in cells [224]. 

PQSs have been identified in the HCV genome, particularly in the 5′ UTR, the core-coding region, and the 3′ UTR [225]. The formation of G4s in these regions has been implicated in the regulation of HCV replication, translation, and virion assembly. In RNA viruses, RdRp is responsible for viral replication [223]. Performing an RNA stop assay with an RdRp-based primer-dependent mechanism showed that PDP, a PDS variant, successfully binds and stabilizes a G4 structure, preventing RdRp from completing replication. Additionally, both PDP and TMPyP4 reduced HCV RNA levels in living cells in a dose-dependent manner [226]. To investigate NCL and viral RNA G4 interaction, PDP competed for G4 binding. Colocalization results showed reduced NCL levels when incubated with PDP [227]. Nonstructural protein 3 (NS3) of HCV contains helicase activity that is essential for viral replication. By utilizing fluorescence anisotropy binding and G4 reporter assays, it has been determined that NS3 can unfold the conserved G4 structures within HCV’s genome and the negative strand. These findings imply that NS3 could hold an important novel role in regulating HCV [228].

### 5.3. Zika Virus and Other Flaviviruses

Zika virus (ZIKV) is a flavivirus transmitted by mosquitoes and shares similarities with other medically significant flaviviruses such as dengue, West Nile, Japanese encephalitis, and yellow fever viruses. While ZIKV infections are often asymptomatic or result in mild illness, recent outbreaks have shown severe neurological complications, including microcephaly in newborns and Guillain–Barré syndrome in adults [229]. Flaviviruses possess a positive-sense RNA genome of about 11 kb with an open reading frame flanked by untranslated regions at the 5′ and 3′ ends. The polyprotein encoded by this ORF is cleaved into three structural proteins (capsid, premembrane/membrane, envelope) and seven nonstructural proteins (NS1, NS2A, NS2B, NS3, NS4A, NS4B, and NS5). PQSs have been discovered within the genomes of various flaviviruses, including ZIKV, DENV, and WNV. It has been suggested that these G4s play a role in regulating viral replication, translation, and virion [230]. ZIKV’s genome contains several PQSs throughout its genes and untranslated regions. In vivo, experiments show that TMPyP4 and BRACO19 stabilize G4s in ZIKV, inhibiting viral growth, genome replication, and protein expression [230]. The effects of these stabilizing ligands are seen in a dose-dependent manner [231].

### 5.4. Influenza 

The Orthomyxoviridae family comprises segmented, negative-sense RNA viruses, also called influenza viruses, and causes the seasonal flu. Influenza viruses cause significant morbidity and mortality worldwide. Influenza A (IAV) and B are responsible for most human infections among the four types of influenza viruses classified based on their structures [232]. Although vaccines and antiviral drugs exist to combat the virus’s spread, they remain a substantial public health concern due to their rapid antigenic drift, leading to drug-resistant strains. The genome of these viruses consists of eight or seven single-stranded segments encoding ten to fourteen proteins depending on the type, with each segment encircled by viral [233].

The transmembrane protease serine 2 (TMPRSS2) protein is an essential element in the life cycles of many respiratory viruses, including influenza and coronaviruses. TMPRSS2 is a membrane-spanning protein that belongs to the type II transmembrane serine proteases (TTSPs) family and is implicated in many physiological and pathological processes [234]. TMPRSS2 cleaves the hemagglutinin component of numerous influenza virus subtypes, a pivotal surface protein for viral entry into host cells [235,236]. The promoter of TMPRSS2 contains a G-rich sequence that forms a G4 structure. Seven benzoselenoxanthene analogs were tested against the IAV, and in vitro experiments demonstrated four analogs to have significant G4-stabilizing effects. These benzoselenoxanthenes effectively stabilized the G4 structure within TMPRSS2’s promoter, resulting in a dose-dependent reduction in TMPRSS2 expression observed in Calu3 cells [237]. Additional G4s have been recognized within the IAV, including regions encoding polymerase complex proteins [238]. Nevertheless, further investigation is imperative to ascertain whether these regions exert a regulatory function on this viral pathogen.

### 5.5. Enterovirus 

Enteroviruses belong to the Picornaviridae family and are positive-sense, single-stranded RNA viruses that cause various human diseases such as myocarditis, meningitis, poliomyelitis, and hand-foot-and-mouth disease [239]. Despite being a significant public health concern, effective antiviral treatments for enteroviruses remain limited. Recent research has identified G4s in enterovirus genomes that regulate viral processes. Targeting these G4s with stabilizing ligands is a promising new approach for combating enterovirus infections. G4 sequences have been detected in the genomes of various enteroviruses, comprising coxsackievirus, poliovirus, and enterovirus 71 (EV71) [107]. G4 formation can potentially influence secondary RNA structures, leading to changes in interactions between viral proteins or host–viral proteins. This, in turn, can impact protein synthesis and overall viral replication [240].

EV71, with its 21 PQSs, exhibited effective inhibition of viral replication using G4-stabilizing ligands, such as BRACO19, PDS, and TMPyP4. This inhibition was demonstrated without compromising cell viability, as evidenced by quantitative PCR data showing a significant decrease in viral RNA levels upon treatment with these ligands. The inhibitory impact of BRACO19 was further validated by plaque assay results, showcasing a substantial reduction in infectivity exceeding an order of magnitude. Beyond replication, BRACO19 has an additional effect on viral translation, specifically reducing the synthesis of the nonstructural protein 2C. In contrast, PDS treatment shows a more modest reduction in the synthesis of this protein [240].

### 5.6. Coronaviruses

The COVID-19 pandemic, caused by the novel coronavirus SARS-CoV-2, has significantly impacted global public health and highlights an urgent need for effective antiviral therapies. This positive-sense, single-stranded RNA virus is responsible for high morbidity and mortality rates worldwide. Despite the rapid development of vaccines, there is a critical need for potent antiviral treatments. Bioinformatic analyses have identified PQSs in specific regions of the SARS-CoV-2 genome, including those encoding proteins and untranslated sections like 5′ and 3′ UTRs, which may influence the secondary structures of viral RNA and impact host–viral protein interactions, similar to other viruses [241,242,243]. Moreover, G4s are located in critical regions such as ORF1ab, 3a, spike (S), nucleocapsid (N), and membrane (M), suggesting their potential involvement in regulating viral replication, assembly, and immune-response modulation [244]. A primer extension assay, utilizing GFP-tagged plasmids with SARS-CoV-2 G4 sequences, demonstrated that G4-stabilizing ligands BRACO19 and TMPyP4 successfully stabilized the G4 structure, preventing primer extension. Transfecting these plasmids into cells, with or without BRACO19 and TMPyP4 treatment, revealed a decrease in GFP expression, highlighting the impact of G4-stabilizing ligands on gene expression [245]. 

The SARS-unique domain (SUD) has been proposed to be crucial for viral transcription and replication [246,247]. There is high homology between the SARS-CoV and SARS-CoV-2 NSP3 SUD proteins [106]. PhenDC3, PDC, phenanthroline derivatives, and metalated porphyrins were subjected to testing to assess their potential interaction with the SARS-CoV-2 SUD. Employing the cellular RNA G4 sequence (TRF2) and employing homogenous time-resolved fluorescence (HTRF), the study revealed that all the mentioned G4-stabilizing ligands effectively inhibited the interaction between SUD and G4. These findings imply a preferential interaction with the host-cell DNA or RNA rather than viral RNA [248].

TMPRSS2 is a highly expressed serine protease in human tissues and is involved in the entry of coronaviruses into host cells [249]. Specifically, SARS-CoV-2 utilizes its S protein to bind to the angiotensin-converting enzyme 2 (ACE2) host-cell receptor. Following binding, the S protein is primed by TMPRSS2 to enable membrane fusion and viral entry into the host cell [249]. Interestingly, PDS, carboxypyridostatin (cPDS), and TMPyP4 bind to a G4 in TMPRSS2, reducing protein levels. Using a pseudovirus system, the vesicular stomatitis virus was pseudotyped with SARS-CoV-2 S glycoprotein (SARS-CoV-2-S-Luc) to determine if stabilizing the G4 in TMPRSS2 affected viral entry. Luciferase activity indicates that PDS, cPDS, and TMPyP4 all reduced pseudovirus entry into cells. Furthermore, utilizing mice that heterogeneously expressed hACE2 via adeno-associated virus (AAV) treated with PDS showed a decrease in VSV-SARS-2-luc infection [250]. In their study, Qin et al. demonstrated the targeting of SARS-CoV-2 G4s by TMPyP4 through experiments conducted on Syrian hamsters and transgenic mouse models. The results indicated that nontoxic levels of TMPyP4 effectively suppressed SARS-CoV-2 infection, leading to reduced viral loads and lung lesions. Moreover, in experimental assessments, TMPyP4 exhibited greater efficacy than remdesivir, a compound that has demonstrated clinical benefits for patient outcomes in trials [251], in both in vitro and in vivo experiments [252]. 

Within the coding region of SARS-CoV-2, a stable RNA G4 structure (RG1) is formed in the N protein. The G4 in the N sequence has been confirmed to be stabilized by PDP, leading to a substantial reduction in SARS-CoV-2 N protein levels. This occurs through the inhibition of N translation in both in vitro and in vivo settings [253]. Oliva et al. showed that berberine, an isoquinoline alkaloid, can bind to and stabilize RG1 in SARS-CoV-2 [254]. However, further investigation in infected cells is required to assess whether berberine effectively reduces SARS-CoV-2 infectivity. Methylene blue (M-Blue), a tricyclic phenothiazine compound, has been shown to have G4-stabilizing properties [255]. M-Blue has been previously approved by the FDA for the treatment of methemoglobinemia and other medical applications [256]. M-Blue has been shown to inhibit the entry of SARS-CoV-2 spike-bearing pseudovirus into ACE2-expressing cells [257]. Qin et al. give an overview of the current advancements in stabilizing G4s in SARS-CoV-2 [258]. They emphasize the need for continual study to advance the progress in targeting G4s as a viable therapeutic avenue. 

The porcine epidemic diarrhea virus (PEDV), a type of coronavirus that affects pigs, has had a severe and widespread impact on the global pig-farming industry. G4s have been confirmed to form in the S and Nsp5 sequences. Confirmed stabilization of the G4 in Nsp5 with BRACO19 and PhenDC3 inhibits replication by a magnitude of 1.5–3.5. PDS and TMPyP4 inhibit both replication and protein synthesis by 99% [259]. This is a great example of different G4 stabilizers having varying levels of viral inhibition. 

## 6. Discussion

The recent exploration of viral G4s and utilizing stabilizing ligands as potential targeting agents present a promising avenue for developing a novel antiviral strategy. The regulatory role of G4s in viral processes such as replication, transcription, and virion secretion has garnered increasing attention. These noncanonical nucleic acid structures, formed in guanine-rich sequences, have been identified in the genomes of various viruses and can be used to target both DNA and RNA viruses. G4s can be used to target viruses directly, such as how BRACO19 can directly stabilize G4 within HIV-1, resulting in a downregulation of viral replication [62]. There is also the opportunity to target host G4s to cause antiviral effects indirectly. As previously mentioned, the host machinery is necessary for viral replication, and there are G4 sites within the gene promoters [54,114,115]. These G4s can be stabilized, causing transcriptional regulation, allowing the cell to be less permissive and increase antiviral activity. There is also the ability to disrupt G4 stabilization to downregulate viral persistence. For instance, gaining insights into the relationship between NCL and KSHV’s LANA and potentially disrupting this interaction could enhance antigen presentation. This, in turn, would allow the host’s immune system to target infected cells more effectively. A strategy based on understanding the relationship between G4s and viruses could potentially lead to virus eradication and the development of innovative therapeutic approaches for treating fatal human diseases. This is a great example of how understanding the regulatory roles G4s play in viral processes can aid in identifying viable therapeutic targets.

However, challenges persist in translating these findings into clinical applications. The typical chemical features shared by many G4 ligands hinder their selectivity, limiting their druggability. Structural characterization of G4 moieties, particularly in the loop and groove regions, is essential for designing more selective ligands, although research in this direction remains limited. However, what is known is that the grooves are involved in forming a compact structure that can impact transcription [260]. Strengthening the potency of G4-stabilizing ligands is imperative to maximize their antiviral potential. Developing antiviral G4 ligands faces hurdles in achieving selectivity for viral G4s or specific cellular G4s. The goal is to minimize the off-target effects. Additionally, there is concern about the toxicity the ligands may have on the host. Interestingly, there is emerging research showing the low-toxicity compound M-Blue has G4-stabilizing properties. Current research on M-Blue surrounds its use in combating SARS-CoV-2. M-Blue contains many qualities ideal for a G4 ligand: low toxicity, stable, inexpensive, and already FDA-approved. Continued research is needed to see if M-Blue is a viable G4 ligand for targeting virial G4s.

To better understand G4 interactions in viruses, NMR is needed. This would allow for insight when designing appropriate G4 ligands. Very few interactions have been determined, such as HIV-1, LTR-III, and LTR-IV [261,262]. Groove interactions between LTR-III’s G4 and the NDI derivative selectivity for the G4 show that this is a promising approach for specific ligand development.

What makes targeting G4s an interesting approach to attenuating viruses can be seen in Figure 4 and Figure 5. Many viruses can be regulated by a single G4 ligand. For example, BRACO19 alone has been confirmed to regulate viral expression for HIV-1, ZIKA, SARS-CoV-2, HSV-1, HPV, MPXV, and VACV. Another ligand, TMPyP4, can inhibit viral processes in HCV, ZIKA, EV71, SARS-CoV-2, HSV-1, HCMV, KSHV, HPV, MPXV, and VACV. It is also important to consider that because G4s are highly conserved, there is the possibility that any variants of these viruses have the potential to be regulated by the same ligands. For example, SARS-CoV-2 has had several variants emerge and cause varying degrees of disease. However, experiments show that BRACO19 and TMPyP4 can both inhibit SARS-CoV-2 replication. Therefore, these G4 ligands can inhibit viral replication for the SARS-CoV-2 variants due to the conserved nature of G4s. These reasons exemplify the need to study G4 interactions and ligand development continually.

## 7. Future Perspective

G4s in Genome Packaging: the intricate orchestration of virus genome packaging involves an intriguing interplay with G4s, introducing a novel dimension to our understanding of viral assembly. Within the viral genome, G4 structures act as specific recognition sites for viral packaging proteins. These proteins, finely tuned to recognize and interact with G4 motifs, play a pivotal role in facilitating the selective packaging of viral genetic material into nascent virions. The dynamic nature of G4 structures adds a layer of complexity to the genome packaging process. The temporal and spatial cues provided by the formation and resolution of G4s likely influence the precise timing and efficiency of genome packaging, contributing to the overall success of the virus life cycle. Furthermore, the interactions between G4s and viral structural proteins extend beyond specific recognition, impacting the structural integrity of the viral capsid or envelope. This influence on stability and morphology, in turn, affects the ability of the virion to protect and package the viral genome effectively. Understanding the nuanced roles of G4s in genome packaging is crucial for developing targeted antiviral strategies and advancing our comprehension of the intricacies involved in the assembly of infectious viral particles.

Unexplored Roles of G4s in the Virus Life Cycle: while the well-established functions of G4s continue to unravel, there are intriguing, yet speculative, roles that remain largely unexplored in the virus life cycle. One such area of interest lies in the potential involvement of G4s in viral RNA editing and evolution. The structural diversity introduced by G4 structures may influence RNA editing mechanisms, impacting the generation of viral quasispecies and contributing to the overall evolution of the virus. Additionally, G4s within the viral genome may interact with host-cell signaling pathways, modulating host responses and potentially aiding in immune evasion. The speculation extends to the contribution of G4s to virus-induced stress granule formation, where these dynamic RNA–protein aggregates play a role in cellular stress responses, creating an environment conducive to viral replication or persistence. Moreover, G4s might play a crucial role in the establishment and maintenance of viral latency, contributing to the stability of the latent viral genome. The resolution of G4s may emerge as a key event in the reactivation process, shedding light on the mechanisms governing viral dormancy. These speculative roles highlight the intricate interplay between G4s and various facets of the virus life cycle, offering a rich landscape for future exploration. Research in these areas may uncover novel therapeutic targets, providing insights into the complex dynamics of virus–host interactions.
